# Application of Interactive and Intelligent Packaging for Fresh Fish Shelf-Life Monitoring

**DOI:** 10.3389/fnut.2021.677884

**Published:** 2021-06-21

**Authors:** Saber Ibrahim, Hager Fahmy, Shimaa Salah

**Affiliations:** ^1^Packaging Materials Department, National Research Centre, Giza, Egypt; ^2^Nanomaterials Investigation Lab., Central Laboratory Network, National Research Centre, Giza, Egypt; ^3^Department of Advertising, Printing, and Publishing, Faculty of Applied Arts, Benha University, Benha, Egypt

**Keywords:** smart packaging, food quality, interactive package, shelf life, Nile perch

## Abstract

Smart packaging, also known as intelligent packaging, is responsive to external stimuli, moisture, light, oxygen, heat, pH, and bacterial growth. It has evolved from extensive applications in food safety, bacterial response, and medical packaging. Interactive packaging has a scientific basis for additional information about food products because these codes give all required data. This work deals with a combination of frontline food sciences, smart and interactive packaging that are applicable for future production of nutrition packages through smart detection of food spooling. Additionally, it verifies the best degree of food safety and population demands as the third generation of packaging technology. High qualified duplex laminated package with a nano-encapsulated pH monitoring label for fresh fish was printed. The interactive Quick Response code icon was combined in a designed package with important information about cooking, smart packaging, and fish quality. Therefore, particle size, zeta potential, and surface area are measured for a nanoencapsulated indicator which exhibits 74.4 nm, 23.6 mV, and 88.9 m^2^/g, respectively: overall migration, water vapor, and oxygen permeability. The properties of printing for 11 color spots are evaluated by x-rite before and after the cold storage period without any detectable changes in the rate of color change (ΔE). The bacterial count of the tested sample is examined by counting the microbial colonies in the agar plate media. There is good agreement between microbial count and smart indicator color change as an effective direct detection tool for sustainable food quality and safety.

## Introduction

Human health has been influenced by the quality of foods and their types. The demands of high food quality products with improved quality and shelf life are on the rise globally. For that, packaging materials play a vital role in saving the food quality, especially the third generation of packaging that indicates the spoiling metabolism of food throughout using smart, responsive materials.

In addition, Packaging functions can be classified into the following protection, communication, containment, and convenience (1). The second generation of packaging involves reducing the environmental footprint of pre-packaged foods (2). Whereas, the third-generation smart packaging can adequately screen the quality of fish products and represent data once changes in food quality occur and caution of potential issues (3). These issues are intended for developing the interest as safer foods with a long shelf life of usability to assure sanitation, detectability, and quality (4). The powerful monitoring of the packaged food status gives data to the nature of the bundled food during transportation and storage (5).

Spontaneously, the interactive packaging design based on Quick Response code (QR) adds another dimension to clarify the utilization of smart packaging plus checking the product shelf life. In this kind of interactive smart packaging, a shrewd outsider gadget, for example, a smartphone, is allocated to examine a coding framework to perceive pictures (6–8) and its site, which gives data like the item smart packaging and “fresh check indicator.”

Freshness indicators could give quick product quality data because of microbial development or synthetic changes inside a food product (9). Encapsulation of responsive materials is the most effective technique and easily applied in industrial production (10). Oxygen, nitrogen, carbon dioxide, moisture, and pH sensors were applied with polymers as supporting materials (11). Following the metabolism process of packaged food has great attention to evaluating nutrient quality and avoiding poisoning accidents (12). This process can be detected by determining gas evolved or pH change with sensory character as color, smell, and forms. The color change of the pH sensor is the easiest and cheapest way to use for food quality control (13). Recently, many research groups deal with pH indicator response with one or more materials through conventional methods, limiting the sensitivity of measurement (13, 14). The novel nanomaterials research field is still in the first step to applying pH sensors as a label. Microbial growth is the key factor in the metabolism of food to be spoiled with pH change (15, 16).

Moreover, microbiological quality might be resolved visually through responses between microbial development metabolites and incorporated indicators and pointers inside the package (17). Most of the freshness indicators depend on the color change of the marker/indicator tag for the presence of microbial metabolites created during the developing microorganisms. All these signs are referring to how the food is still new or not (18). Freshness indicators can likewise be utilized to gauge the rest of the timeframe of realistic usability of perishable items (19). Although the use of QR codes in the packaging products has been applied already, the integration of “interactivity” and “smartness” into a similar packaging design is a significant additional worth and new trend in the showcasing advantage (20, 21). Thus, interactive QR to detect smart monitoring of shelf life of fresh fish-based products is an innovative value. It can provide a new dimension in packaging quality, saving product waste, righteous monitoring of the product shelf life, and better communication with the customers (22). Therefore, the research problem is about the inability of the consumer to evaluate the chemical, biological, and physical processes, leading him to throw the product if any slight change appeared. However, it is still usable and consequences unintended waste of the product (23). On the contrary, it could lead to using the expired product, with a false expiration date of the product which causes poisoning and deaths (24).

This paper, a smart and interactive package as the third generation of packaging, was designed and printed with a novel nanoencapsulated pH indicator as a color response label. The reverse printing is performed and applied on polyester/polyethylene duplex laminated structure material (PET 12/ PE 40 micron) using a polyurethane adhesive. The microbial population was followed over 288 h and compared with label color transformation as a direct tool for food quality by bacterial population detection. Combining multidiscipline frontier sciences, nanotechnology, smart packaging, and interactive packages has been enhanced for food quality detection to one of abundance food types. The fresh fish filet was kept in storage for over 12 days at 4°C with follow-up from microbial growth and pH response using a validation indicator.

## Experimental

### Materials

The interactive package design was constructed in the Adobe Illustrator CC 2018 program in cyan, magenta, yellow, and black (CMYK) colors with spot colors. Processing machine photopolymer flexo-plate ESKO was used to produce the flexo photopolymer plate thickness 1.14 mm, Germany. The interactive package was printed on flexographic machine supplier Bobst 8 color 2019, Germany. The reverse printing was applied on polyester/polyethylene duplex laminated structure material (PET 12 μm/PE 40 μm) with polyurethane solvent-less adhesive. The rate of color change ΔE was measured with X-Rite exact, Pantone, the USA for the printed packages.

Lactic acid, stannous octanoates, and methyl red are purchased from Sigma-Aldrich, Germany. Additionally, chloroform, acetone, and ethanol were distilled and kept over calcium hydrate until used. Nile perch fishes were purchased from the Egyptian food market (Cairo) and were prepared as a filet with direct packaging in a printed duplex smart and interactive package. The nutrient agar (Lab M Limited, Heywood, Lancashire BL9JJ, United Kingdom) was used, and the potato dextrose agar (Ponadisa, Laboratories Conda S.A, Madrid, Spain) was purchased.

### Synthesis of Nanoencapsulated

According to our previous work, with little modification, the synthesis of polylactic acid and encapsulation reaction of methyl red occurred with little modification (25). Methyl red was dissolved in ethanol (0.05 mol. L^−1^). The 20 ml of methyl red solutions was mixed with (0.21 mol) lactic acid monomer. The mixture was charged in a three-neck round bottom flask connected with vacuum and nitrogen inlets. The reaction flask was immersed in a temperature-controlled water bath during nitrogen purge for 1 h with stirring 800 rpm. The temperature was raised gradually up to 75°C. The polymerization initiator 2.1 × 10^−2^ mol of stannous octanoates was added dropwise over 15 min. The reaction was continued for 10 h under vacuum. After that, the reaction was immersed in an ice bath, and the prepared methyl red/polymer was precipitated in acetone through a dropwise separation funnel. The precipitate was dried under vacuum at 50°C for 24 h. After dying, the indicator circle was cast in a circle Teflon plate and drying in a vacuum oven at 40°C for 24 h.

### Smart Packaging Film Formation

The prepared polymer/methyl red nanoencapsulation was dissolved in chloroform with solid content 20 wt% and mixed for 30 min over a magnetic stirrer. Then, PLA/pH indicator solution was purged with nitrogen for 30 min and immersed in a sonication bath for 10 min to remove any air bubble. The solution was cast in a Teflon Petri dish and dried in a vacuum oven overnight at 40°C. The film was cut into small round pieces to be used as indicator pads. All pads were attached to the inner side of the duplex package film by pressing under a mechanical hydrolytic piston at 80°C for 2 min.

### Measurements and Analysis

#### Investigation of Smart Nanoencapsulated Polymer Composite

The mean diameter and zeta potential of the nanoencapsulated particles were determined at 170 and 18°, respectively, by dynamic light scattering (DLS) (NICOMP 380 ZLS, PSS, Santa Barbara, CA, USA). Nitrogen adsorption-desorption measurements were carried out at 77.35 K on a Nova Touch LX^4^ Quantachrome Instruments, Florida, USA, to determine the Brunauer–Emmett–Teller (BET) surface area. All samples were kept dry in a desiccator until testing. Samples were cooled with liquid nitrogen and analyzed by measuring the volume of gas (N_2_) adsorbed at specific pressures. The pore volume was taken from the adsorption branch of the isotherm at P/P_o_ = 0.995, assuming complete surface saturation.

Water vapor transmission rate (WVTR) was performed using GBI W303 (B) Water Vapor Permeability Analyzer (Labthink Instruments Co. Ltd., Jinan, China) *via* the cup method. The water vapor permeability was measured as the amount of water vapor passes through the tested film. Also, WVTR was measured as the mass of water vapor transmitted throughout a unit area in a unit time under controlled conditions of temperature (38°C) and (90% relative humidity, RH) according to the following standards (ASTM E96, ISO 2528, ASTM D1653, TAPPI T464, DIN 53122-1, JIS Z0208). Also, the oxygen transmission rate (OTR) under conditions (23°C and 50% RH) was measured by N530 Gas Permeability Analyzer (Labthink Instruments Co. Ltd., Jinan, China) according to the following Standards ISO2556-2001, GB/T1038-2000, ASTM D1434-82(2003).

#### Overall Migration Test

Many international regulations have been legalized regarding the migration of specific substances such as heavy metals, degradation products, and additives. These materials could have bad taste, odors, or suspected harmful effects on consumers. The OM simulants and conditions as detailed in the European Union (EU) Regulation are categorized in Nr. 10/2011 (The PIM), Simulant A, Simulant B, Simulant D2. All samples were compared with a blank sample (Millipore water with resistivity 18.5 MΩ) as a reference. The OM is expressed as the amount in milligrams of material lost from one decimeter square surface (mg/dm^2^). OM results were calculated according to (EN 1186-5-single side contact in cell test) (26).

#### Microbiological Evaluations

The microbial infection of the tested sample was examined by counting the microbial colonies in the agar plate media (CFU agar plate method). Mixed sample with sterilized bi-distilled water (0.1 g/ml w/v) and then transferred aseptically to a stomacher bag and shaking for 4 h at 180 rpm. Serial dilutions (10^−2^–10^−6^) were performed, and dilutions were plated in duplicate. The spread plate and pour plate methods enumerated total aerobic bacteria (at 37°C for 2 days). In comparison, yeasts and molds (at 28°C for 3 days) were performed on the nutrient agar (Lab M Limited, Heywood, Lancashire BL9JJ, United Kingdom) and the potato dextrose agar (Ponadisa, Laboratories Conda S.A, Madrid, Spain), respectively. The microbial colonies were counted and expressed as CFU/g of the tested sample (27).

## Results and Discussion

### Investigation of Smart Nanoencapsulated

**Figure 1:**
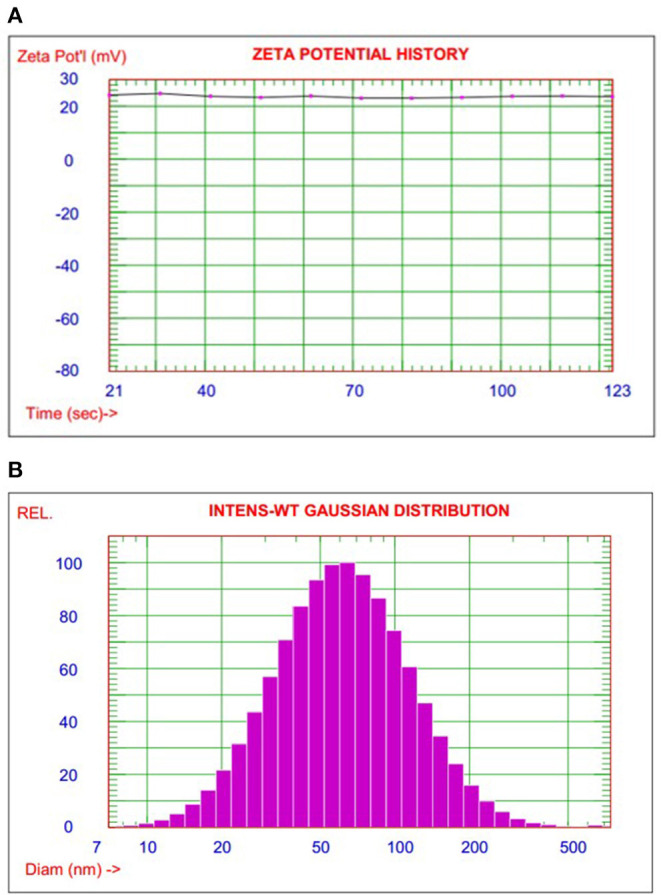
**(A)** The particle size distribution and zeta potential measurement for nanoencapsulated smart polymer composites. **(B)** Illustrated the relation of pH value with the color change of smart package label.

The particle size test is one of the high considered measurements to evaluate the efficiency of the preparation method to fulfill the target materials properties. DLS examined nanoencapsulated smart food responsive composite to investigate the particle size distribution as shown in Figure 1. The average particle size of nanocomposite smart materials was 70 nm with a range of 10–500 nm. The polydispersity index (PDI) was indicated to narrow particle size variation 0.21 as a unimodal bell shape of particle diameter homogenized distribution.

Zeta potential measurement was calculated as an average value for a sequence of measurements, as shown in Figure 1. The average zeta potential is 23.9 mV which indicated good dispersion with nice stabilization of nanoencapsulated particle. These results can strongly recommend nanoencapsulated smart, responsive polymer to form homogenized smart polymer film by simple casting techniques.

On the other hand, adsorption/desorption isotherm measurements give a high surface area of 88.9 m^2^/g with an excellent correlation coefficient of 0.996 as a first-order fitting relationship which indicated good efficiency and accuracy of calculated BET value. Overall, there is a high degree of agreement between particle size and surface area measurements as indirect proportional relationships (28).

### Smart Interactive Design and Printing Properties

**Figure 2:**
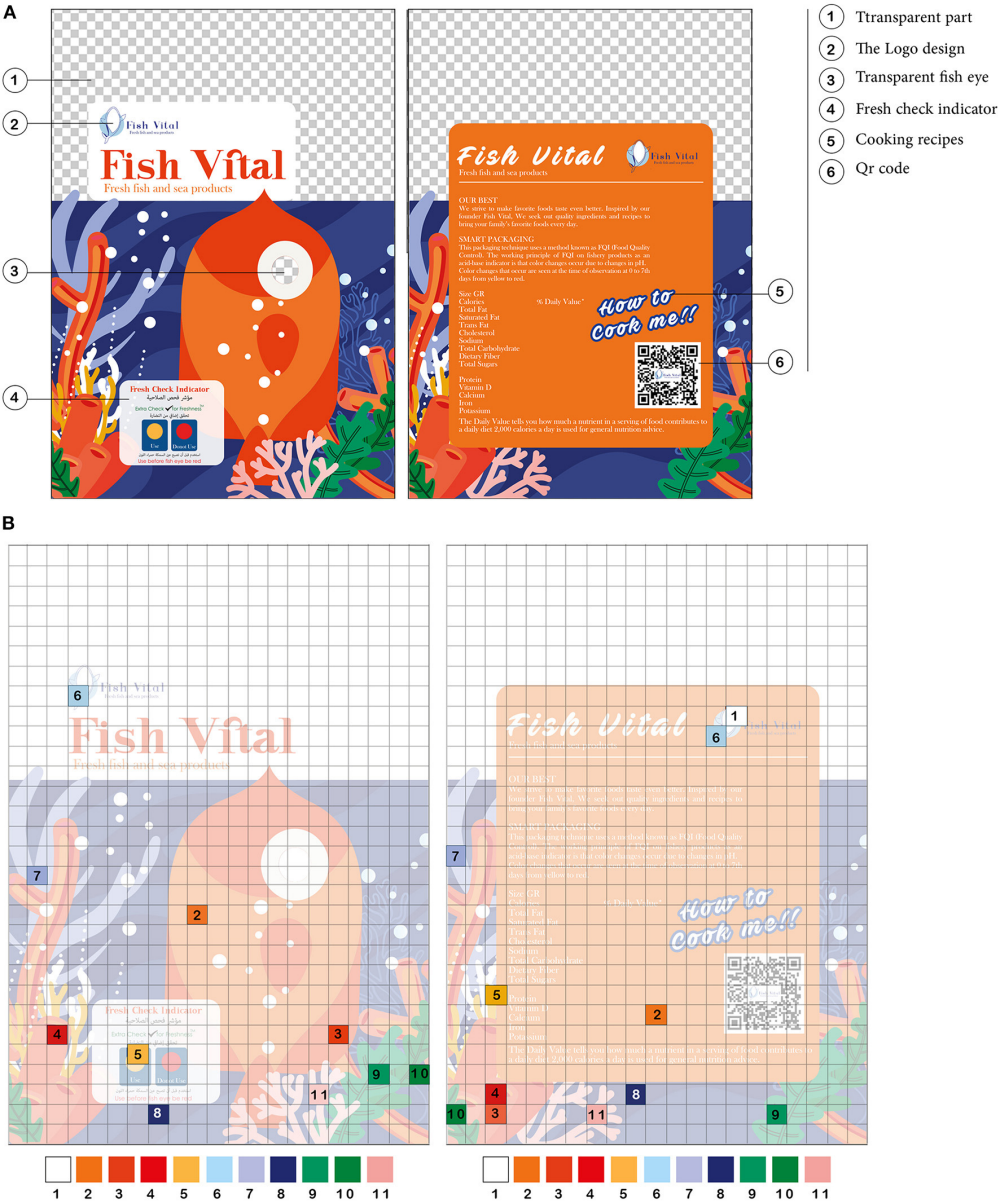
**(A)** The designed interactive package and **(B)** the color spot on the package.

The contrast color was used in an interactive design to attract consumer attention, as shown in Figure 2A. The transparent part at the top of the design has shown the fresh fish products. Nanoencapsulated indicator film was combined with a package in eye fish design to detect the color change. QR code was applied in the interactive package to represent the product website *via* smartphone with product information.

Additionally, polyester (PET)/polyethylene (PE) film was reversely printed through the flexographic process and laminated as a high barrier interactive package with excellent durability, environmental stability, and mechanical flexibility characters of used nitrocellulose inks (2). The PET film has known properties as thermal stability, tensile resistance strength, and excellent gas and moisture barrier properties. Moreover, PE film contributes as an inner package welder and barrier layer between printed inks and fish filets as inert protecting layers (29).

The rate change of colors (ΔE) for the printed package before and after cool storage for 12 days at 4°C. Eleven color spots were selected to be measured with X-Rite, as shown in Figure 2B, and the results were inserted in Table 1.

**Table 1:** Measurement of 11 color spots on the package before and after cooling storage.

	**REF^#^**	TEST^*^	**ΔE**	**REF^#^**	**TEST**	**ΔE**	**REF^#^**	**TEST**	**ΔE**	**REF^#^**	**TEST**	**ΔE**
	**Color** 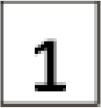			**Color** 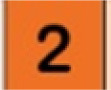			**Color** 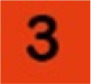			**Color** 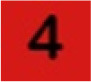		
L	74.27	−0.51	0.41	49.30	0.56	1.10	39.08	0.63	0.58	35.69	0.34	0.29
A	−3.24	0.06		33.13	−0.66		47.22	0.21		58.49	0.27	
B	−7.12	−0.21		38.78	−2.36		28.32	−0.22		30.24	0.08	
	**Color** 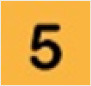			**Color** 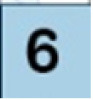			**Color** 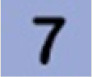			**Color** 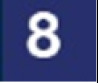		
L	54.42	0.45	0.67	63.75	0.42	0.37	46.93	0.80	0.79	19.54	0.74	0.52
A	16.82	0.73		−12.95	0.12		0.36	0.08		7.30	−0.33	
B	38.42	−0.04		−20.70	−0.14		−22.87	0.79		−38.58	0.42	
	**Color** 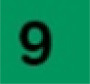			**Color** 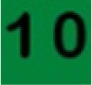			**Color** 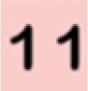					
L	43.87	43.87	0.59	37.96	0.11	0.41	51.27	0.28	0.28			
A	−51.58	1.45		−30.35	0.51		25.26	−0.06				
B	7.35	−0.83		0.51	−0.82		6.32	−0.08				

# *Reference sample before cooling storage printed film*.

* *Tested sample after cooling storage printed film*.

Differences in ΔE (Δ1.10–Δ0.29) for color spots before and after cool storage were tiny. The higher and lower values of Δ1.10 and Δ0.29 were related to color 2 (orange) and color 4 (red). All measurements are within the permissible limits according to ISO 12647-6. The colors of the printing package were not affected through cool storage as customer demand. This is verified the aim of reverse printing technology to protect the color of the printing area (30).

### Evolution of Packaging Film

#### Oxygen and Water Vapor Permeability

The barrier properties of laminated packaging film were evaluated against oxygen gas and water vapor at 23°C as permeability for the film sector. According to Robertson (29), the transmission of gases through a packaging material can occur through two mechanisms: pore effect and solubility-diffusion effect. In the first case, the gases cross the material by passing through small pinholes or ruptures in the structure. While, in the second case, the concentration difference between the two sides of the packaging material and the solubility of gases in the corresponding material determines the level of transmission (31).

The oxygen transmission rate (OTR) shows excellent barrier property with low oxygen gas permeability with a range of 0.047–0.252 mL m m^−2^ day^−1^ Pa^−1^ at a humidity of 50%. In addition, the water vapor transmission rate was exhibited high barrier properties for water vapors as a successful anti-moisture packaging film. The measured values are 0.351–2.072 g m m^−2^ s^−1^ Pa^−1^ at water vapor saturation of 80%. The water permeability in this study is lower than PE and PET films in the literature which can be discussed to increase the hydrophobicity of the laminated film with an adhesive layer between the two films (32).

#### OM Test of Food Package

Laminated polymer films are materials consisting of multiple layers of different polymers types. These materials are ubiquitous in daily life, with substantial food and pharmaceutical packaging containing laminates. A common requirement of food packaging is that the internal layer must appropriate for contacting food, and the external layer must be suitable for printing product information.

The migration of low molecular weight polymer or chemical additives substances from the bulk polymers laminated film has been studied according to the EU Regulation Nr. 10/2011. The stimulants were selected carefully to represent the different food natures. As shown in Table 2, the duplex laminated PE/PET packaging film shows acceptable migration limits. The OM from the package was ranged from 0.3 up to 1.3 mg/dm^2^ with limits of accepted level up to 10 mg/dm^2^, as shown in Table 1.

**Table 2:** The overall migration from polyester-/polyethylene-laminated duplex packaging film.

**Method**	***EN-1186-5* (simulant A) mg/dm^**2**^**	***EN-1186-5* (simulant B) mg/dm^**2**^**	***EN-1186-5* (simulant C) mg/dm^**2**^**	***EN-1186-4* (simulant D_**2**_) mg/dm^**2**^**
**Replicates**				
1	0.3	0.6	1.1	0.0
2	0.2	0.7	1.3	0.0
3	0.4	0.8	1.4	0.0
Mean result	**0.3**	**0.7**	**1.3**	**0.0**
Limit	**10.0**	**10.0**	**10.0**	**10.0**

*Bold values are the average of results and the limit of measurement*.

#### Microbiological Evaluations

The microbial action of different microbes mainly causes spoilage of fresh and lightly preserved fish products. Therefore, fish products can be spoiled due to the growth of aerobic bacteria, yeasts, and molds. Although the spoilage of fresh fish is well-understood, the spoilage of cool preserved fish products still lacks knowledge. The production of trimethylamine (TMA) with off-odor detection was used as qualitative measurements of microbial spoilage activity (33). On average, cells of *Photobacterium phosphoreum* are found in spoiled packed cod suggesting this organism could be responsible for spoilage (34). A high concentration of *Shewanella putrefaciens* is required in producing detectable off-odors and concluded up this organism without importance for spoilage of packed cod (35).

**Figure 3:**
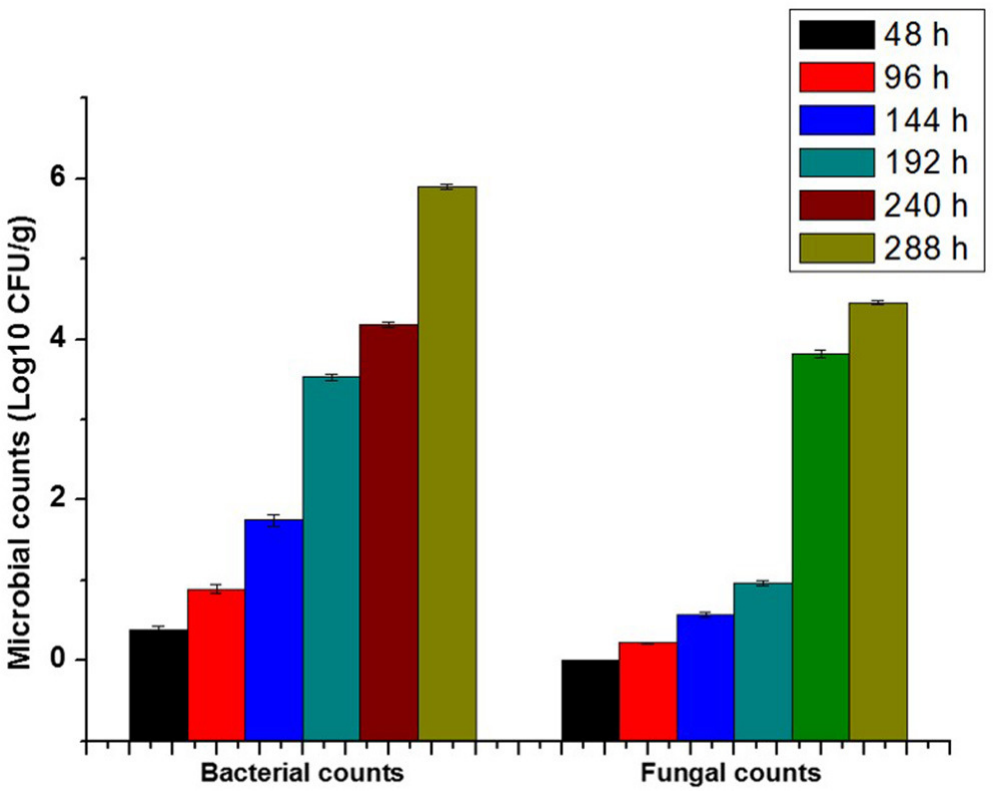
Microbial population over 12 days for cooling fresh fish filet.

The microbial population of bacteria and fungi were illustrated in Figure 3. The microbial growth was increased with geometric propagation up to 10^−7^ and 10^−8^ after 10 and 12 days, respectively. As a rule, fish spoil more rapidly than meat under chill conditions (36). The pH of mammalian muscle, around 5.6, is lower than that of fish (6.2–6.5), contributing to the long storage life of meat. For these reasons, the detection of microbial growth in fish is highly recommended.

**Figure 4:**
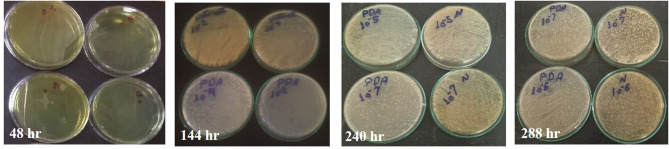
Petri dish photos of microbial activity assay of fresh cooling fish filet with different concentrations (μg/mL) over different incubation time 48, 144, 240, and 288 h.

**Figure 5:**
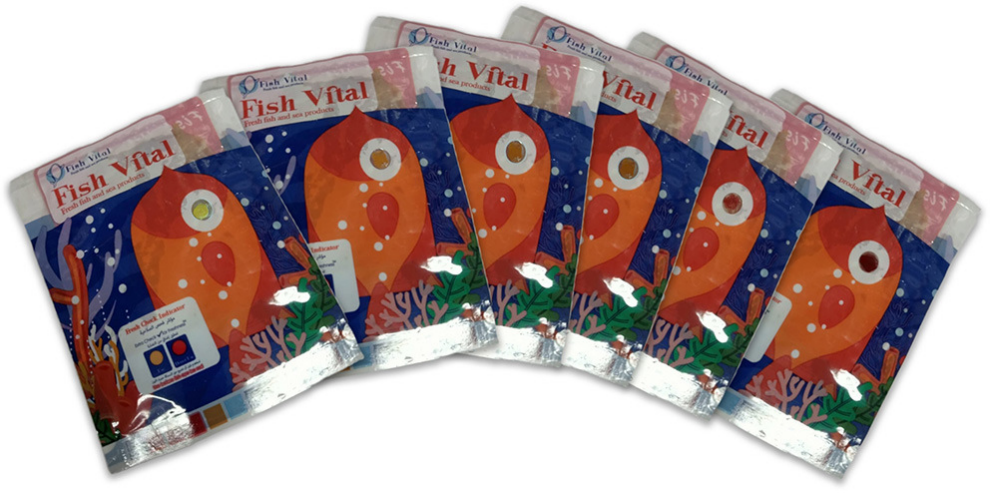
The smart packages during the storage period of fresh fish filet.

The microbial growth after 48 h. was not detected. After that, the population of bacterial and fungal colonies appeared from 96 h until 288 h, as shown in Figure 4. The metabolite of microbial activity is directly related to change in pH value, which acts as a spoiling indication with smart packages, as shown in Figure 5.

The color of the nanoencapsulated indicator in the smart package was changed from yellow to red over six color degrees, as presented in Figure 5. The pH value was increased in direct proportion with microbial population that gives an accurate determination of the food quality over the storage period to avoid any harmful or poisoning accident.

Overall, the initial microbial population was determined immediately after fish packaging with time intervals (for each 48 h). The total aerobic bacteria, the total yeast, and molds were reached the lower value even after 96 h, which became neglected compared with the unpackaged sample. After 6 days of storage, yeasts, molds, and total aerobic bacteria counts were initially increased. It was observed that the total yeast and molds were remarkably lower than that observed in the total aerobic bacteria along with the storage time. Indeed, the packaging of fresh fish in the smart package showed the highest preserved against microbial population *via* reducing the total aerobic bacteria and the total level of yeast and molds over 6 days of storage at 4°C.

## Conclusion

Excellent microbial growth detection was monitored through a developed nanoencapsulation technique for pH sensor materials to evaluate fresh fish quality tools. This research-based one combined multidiscipline science, packaging design as an interactive package, nanomaterials stabilization in polymer matrix through encapsulation process, and microbial evaluation for food quality until spoiling. Package design was successfully printed and combined with a nanopolymer-encapsulated methyl red indicator label. The interactive design provided additional value to the user as it facilitated the monitoring of the fresh fish shelf-life. Microbial population study was concluded with the superb microbial growth detection of packaged fresh fish in pH change until 10^−7^ and 10^−8^ dilution. Also, the microbial growth for bacteria, molds, and fungi was directly related to pH change due to the metabolism process for fresh fish filets. Overall, effective microbial spoiling metabolism was well directly detected through a smart and interactive package with nanoencapsulated color change pH sensors from yellow to red as an efficient motoring tool to verify the validity of fresh fish to use or discard.

## Data Availability Statement

The original contributions presented in the study are included in the article/supplementary material, further inquiries can be directed to the corresponding author.

## Author Contributions

All authors listed have made a substantial, direct and intellectual contribution to the work, and approved it for publication.

## Conflict of Interest

The authors declare that the research was conducted in the absence of any commercial or financial relationships that could be construed as a potential conflict of interest.
